# Stereotactic Body Radiation Therapy Reirradiation for Locally Recurrent Rectal Cancer: Outcomes and Toxicity

**DOI:** 10.1016/j.adro.2020.07.017

**Published:** 2020-08-07

**Authors:** Thomas Smith, Sean M. O’Cathail, Sabrina Silverman, Maxwell Robinson, Yatman Tsang, Mark Harrison, Maria A. Hawkins

**Affiliations:** a Mount Vernon Cancer Centre, East and North Herefordshire NHS Trust, Middlesex, UK; b Institute of Cancer Sciences, University of Glasgow, Glasgow, UK; c Oxford University NHS Foundation Trust, Oxford, England, UK; d Medical Physics and Biomedical Engineering, University College London and University, London, England, UK; e College London Hospitals NHS Foundation Trust, London, England, UK

## Abstract

**Purpose:**

Stereotactic body radiation therapy (SBRT) has emerged as a potential therapeutic option for locally recurrent rectal cancer (LRRC) but contemporaneous clinical data are limited. We aimed to evaluate the local control, toxicity, and survival outcomes in a cohort of patients previously treated with neoadjuvant pelvic radiation therapy for nonmetastatic locally recurrent rectal cancer, now treated with SBRT.

**Methods and Materials:**

Inoperable rectal cancer patients with ≤3 sites of pelvic recurrence and >6 months since prior pelvic radiation therapy were identified from a prospective registry over 4 years. SBRT dose was 30 Gy in 5 fractions, daily or alternate days, using cumulative organ at risk dose constraints. Primary outcome was local control (LC). Secondary outcomes were progression free survival, overall survival, toxicity, and patient reported quality of life scores using the EQ visual analog scale (EQ-VAS) tool.

**Results:**

Thirty patients (35 targets) were included. Median gross tumor volume size was 14.3 cm^3^. In addition, 27 of 30 (90%) previously received 45 to 50.4 Gy in 25 of 28 fractions, with 10% receiving an alternative prescription. All patients received the planned reirradiation SBRT dose. The median follow-up was 24.5 months (interquartile range, 17.8-28.8). The 1-year LC was 84.9% (95% confidence interval [CI], 70.6-99) and a 2-year LC was 69% (95% CI, 51.8-91.9). The median progression free survival was 12.1 months (95% CI, 8.6-17.66), and median overall survival was 28.3 months (95% CI, 17.88-39.5 months). No patient experienced >G2 acute toxicity and only 1 patient experienced late G3 toxicity. Patient-reported QoL outcomes were improved at 3 months after SBRT (Δ EQ-VAS, +10 points, Wilcoxon signed-rank, *P* = .009).

**Conclusions:**

Our study demonstrates that, for small volume pelvic disease relapses from rectal cancer, reirradiation with 30 Gy in 5 fractions is well tolerated and achieves an excellent balance between high local control rates with limited toxicity.

## Introduction

Locally recurrent rectal cancer (LRRC) is defined as recurrence of rectal cancer within the pelvis after previous surgical resection.^1^ Local recurrence rates remain approximately 10% and the majority of those that recur will have received neo adjuvant (chemo) radiation therapy as part of initial multimodality treatment.^2^, ^3^, ^4^ Prognosis is poor with significant morbidity and poor quality of life from pelvic pain, fistula, bleeding, and fecal discharge.^5^ Untreated LRRC has a median survival of 6 months, improving with chemotherapy or radiation 12 to 16 months.^6^

Curative resection remains the most important factor for survival.^6^^,^^7^ However, surgery for LRRC is challenging owing to altered anatomic planes and tissue fibrosis from previous radiation therapy and primary resection. Pelvic exenteration is associated with significant morbidity and not always feasible.^8^^,^^9^ There is also no clear consensus about which cases should go for resection.

Reirradiation is emerging as a therapeutic alternative and has been shown to have a significant palliative effect and favorable survival outcomes.^6^^,^^10^ Delivery is challenging because of concern tissues may have already received doses near the organ tolerance during primary treatment, particularly the small intestine. Historically, LRRC was treated with large volumes to ensure target coverage and planned conformally.^11^^,^^12^ Doses were hyperfractionated (1.2 Gy-1.5 Gy), which had a sound radiobiologic potential to reduce late effects. Additional technical advances, such as the use of IMRT,^13^ have been used to reduce side effects. Proton beam therapy also has a dosimetric advantage compared with photons, with pelvic bone marrow particularly spared in a recent series,^14^ although doses were again hyperfractionated. Stereotactic ablative radiation therapy (SBRT), which involves very accurate delivery of a high radiation dose in a small number of fractions to a target with narrow margins,^15^ can potentially increase the precision and reduce off target effects, in reirradiation cases.^16^

The evidence base to date for the use of SBRT in reirradiation in rectal cancer is limited with just 3 published cohorts, each less than 20 patients.^10^^,^^17^, ^18^, ^19^ The aim of this work is to evaluate the local control, toxicity, and survival outcomes in a contemporaneous cohort of nonmetastatic LRRC patients, treated with SBRT.

## Methods and Materials

### Patient selection

Using a prospectively maintained database, patients treated with reirradiation using SBRT for LRRC at Oxford Cancer and Haematology Centre and Mount Vernon Cancer Center between October 2015 and June 2019 were identified. Patients were previously treated with (chemo) radiation to the pelvis for rectal cancer. Full restaging of both the local and distant disease (computed tomography or magnetic resonance imaging, or positron emission tomography imaging) was mandated before treatment. Patients were all discussed at a colorectal specialist multidisciplinary meeting for surgery and only inoperable patients, with less than 3 metastases, who had a disease-free survival of at least 6 months, World Health Organization performance status 0 to 2, and greater than 6 months since previous radiation therapy were considered suitable for SBRT reirradiation. Where the history, examination, and imaging findings were in keeping with rectal cancer recurrence a repeat biopsy was not required. All patients consented to collection of data as part of enrolment in the SBRT treatment program,^20^ which had received ethical approval (North East – York Research Ethics Committee REC reference: 16_NE_0285).

### Reirradiation

Patients were treated either using a linear accelerator SBRT platform with daily image guided verification with cone beam computed tomography scan (n = 16) or with the Accuray Cyberknife treatment system (n = 14) using fiducial markers or bony landmarks for set up and intrafraction motion management. In addition, 30 Gy in 5 fractions with daily or alternate day fractionation for 7 to 10 days were delivered. The locations of the pelvic recurrences were mapped using a previously published atlas.^21^ To allow equal comparison across the different initial fractionations the EQD2_(α/β 3)_ for late responding tissues and EQD2_(α/β 10),_ BED_10_ for tumors were calculated.

The planning target volume was the gross tumor volume as identified on magnetic resonance imaging with a 3 mm margin in all directions. Organs at risk (OARs) delineated included small and large bowel, bladder, cauda-equina, and sacral plexus or roots. Previous radiation therapy plans and dose volume histograms were reviewed for relevant dose metrics. Dose constraints to OARs overlapping target were prioritized (Table E1; available online at https://doi.org/10.1016/j.adro.2020.07.017), with maximum cumulative doses to small bowel and bladder of 98 Gy_3_ EQD2 and 120 Gy_3_ EQD2, respectively.^22^ For sacral nerve roots, where relevant, recovery of up to a third of previously received doses was assumed. Cumulative dose was calculated using a summation method where addition of the maximal point dose with the OAR from the planning data of the original plan is added to the maximal point dose within the corresponding OAR on the reirradiation plan simulation. No patients received concurrent systemic therapy at the time of SBRT.

### Response and toxicity assessment and follow-up

Treatment response was evaluated according to the response evaluation criteria in solid tumors.^23^ Toxicity within the first 3 months after SBRT was considered acute and late if first occurring after this time point. Clinical or telephone assessments were performed at baseline, 1, 3, and 6 months after SBRT, then at 6 monthly intervals thereafter and imaging at the same timepoints. Toxicity was recorded according to the National Cancer Institute Common Terminology Criteria for Adverse Events, version 4.0. Quality of life was assessed using the EuroQol EQ-5D tool, which consists of the 5 level EQ-5D descriptive system and the EQ visual analog scale (EQ-VAS).^24^ The latter patient reported outcome measure measured on a continuous scale, where 100 signifies “best imaginable health” and 0 “worst imaginable health,” on which patients provide a global assessment of their health.

The primary outcome of interest was local control (LC), defined as recurrence or progression within the reirradiation field. Secondary outcomes were time to first site of radiologic progression (progression free survival [PFS]), time to death (OS), and quality of life outcomes. Locoregional progression was defined a disease progression outside the planning target volume (PTV) but within the pelvis, distant progression as disease sites outside the pelvis.

### Statistical analysis

Median follow-up was ascertained by reverse censoring on death. The cumulative probabilities of LC, PFS, locoregional progression, and OS were calculated using the Kaplan-Meier method and were all defined from time of SBRT until the corresponding event or censored at date of last follow-up. Wilcoxon signed-rank test was used to assess change in EQ-VAS scores over time. All analyses were performed using R studio.

## Results

**Figure 1:**
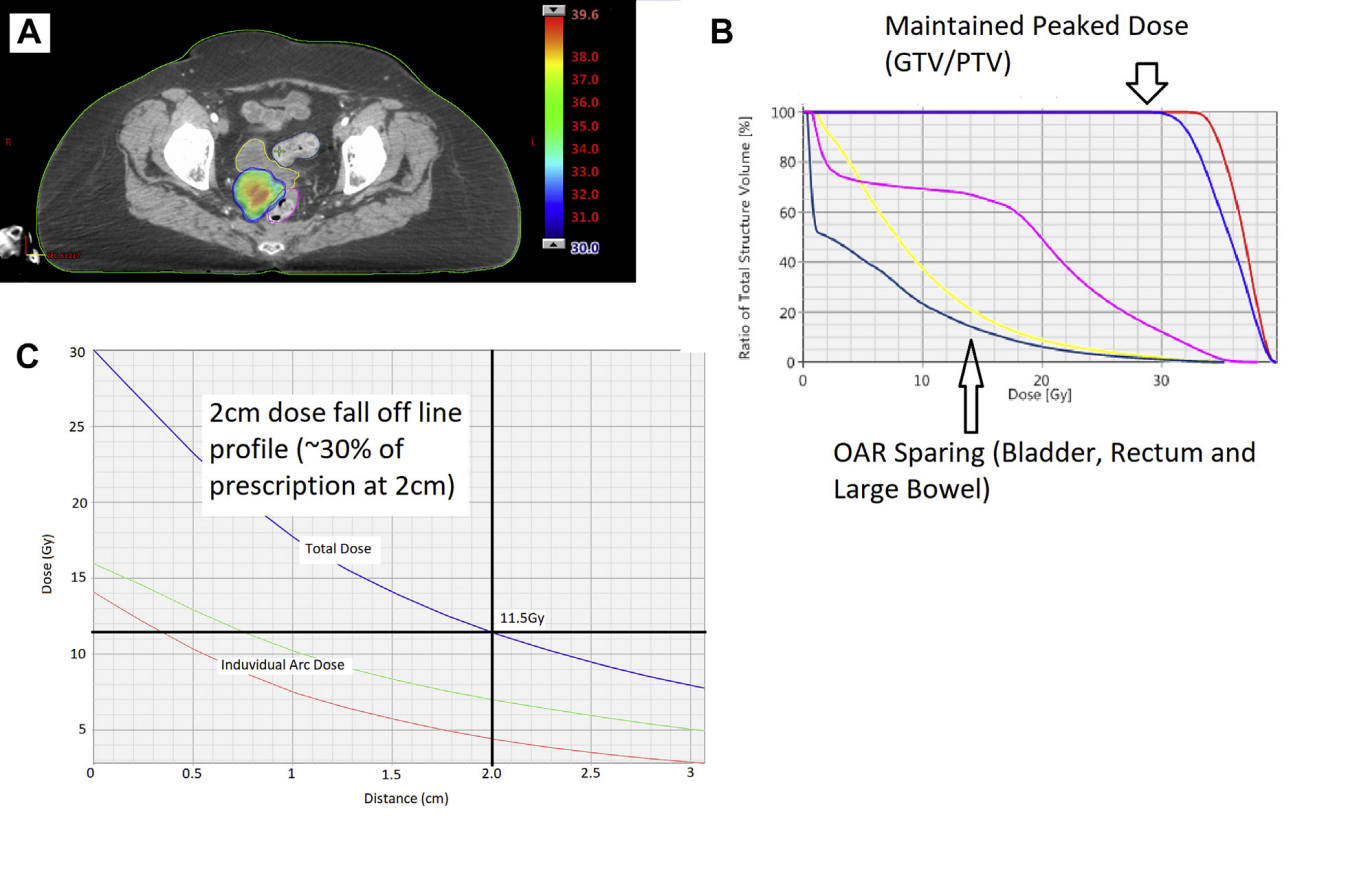
(A) Thirty Gy prescription dose color wash of example case showing irradiation of bilateral pelvic nodal recurrences and resulting dose volume histogram. (B) Thirty Gy prescription dose color wash of single central recurrence and resulting dose volume histogram (C) Example of line profile of dose fall off from the edge of PTV showing rapid dose fall off to 2 cm distance. *Abbreviation*: GTV = gross tumor volume; OAR = organs at risk; PV = planning volume.

Thirty patients, with 35 pelvic metastatic lesions, who met the prespecified inclusion criteria, were included in the analysis. The median follow-up for the whole cohort was 24.5 months (interquartile range, 17.8-28.8). Patient and treatment characteristics are summarized in Table 1. All patients received the intended dose of 30 Gy in 5 fractions and the OAR constraints were respected in all cases. Two examples plans are provided (Fig. 1). Approximately equal numbers were treated using Cyberknife^14^ and volumetric arc therapy linac.^16^ All patients had previously histologically confirmed adenocarcinoma of the rectum and had radical total mesorectal excision of their pelvic primary. In addition, 97% (29/30) had neoadjuvant pelvic radiation therapy with 90% (27/30) receiving chemoradiation and 2 patients receiving short course radiation therapy. One patient received 66 Gy in 33 fractions for prostate bed and had an anterior resection alone for his rectal cancer. Serum prostate specific antigen and prostate-specific membrane antigen positron emission tomography scans testing ruled out prostate cancer recurrence. The interval between completing initial radiation therapy and SBRT reirradiation was a median 41.9 months (range, 11.3-90.4).

**Table 1:** Cohort characteristics

Variable	N = 30	%
Age	65 (range, 36-84)	_
Sex		
Male	19	63
Female	11	37
ECOG		
0	23	77
1	7	23
Treated site		
Lymph node	29	96
Other∗	1	4
Median GTV (range)	14.3 cm^3^ (0.28-89.7)	
Median GTV equivalent diameter (range)	3 cm (0.8-5.54)	
Prior chemotherapy		
Yes	21	70
No	9	30
No. of metastases		
1	25	83
2	5	17
Previous RT dose		
45 Gy/25#	22	73.3
50.4 Gy/28#	4	13.3
50 Gy/25#	1	3.3
66 Gy/33#	1	3.3
25 Gy/5#	2	6.6
Median cumulative dose (range)		
BED_5_	127.2 (116-158.4)	
BED_10_	101.1 (85.5-127.2)	
EQD2_(α/β 10)_	84.2 (71.2-106)	
EQD2_(α/β 3)_	97.2 (94-120)	

*Abbreviations*: ECOG Eastern Cooperative Oncology Group; GTV = gross tumor volume; RT = radiation therapy.

∗ Other = penile bulb.

**Figure 2:**
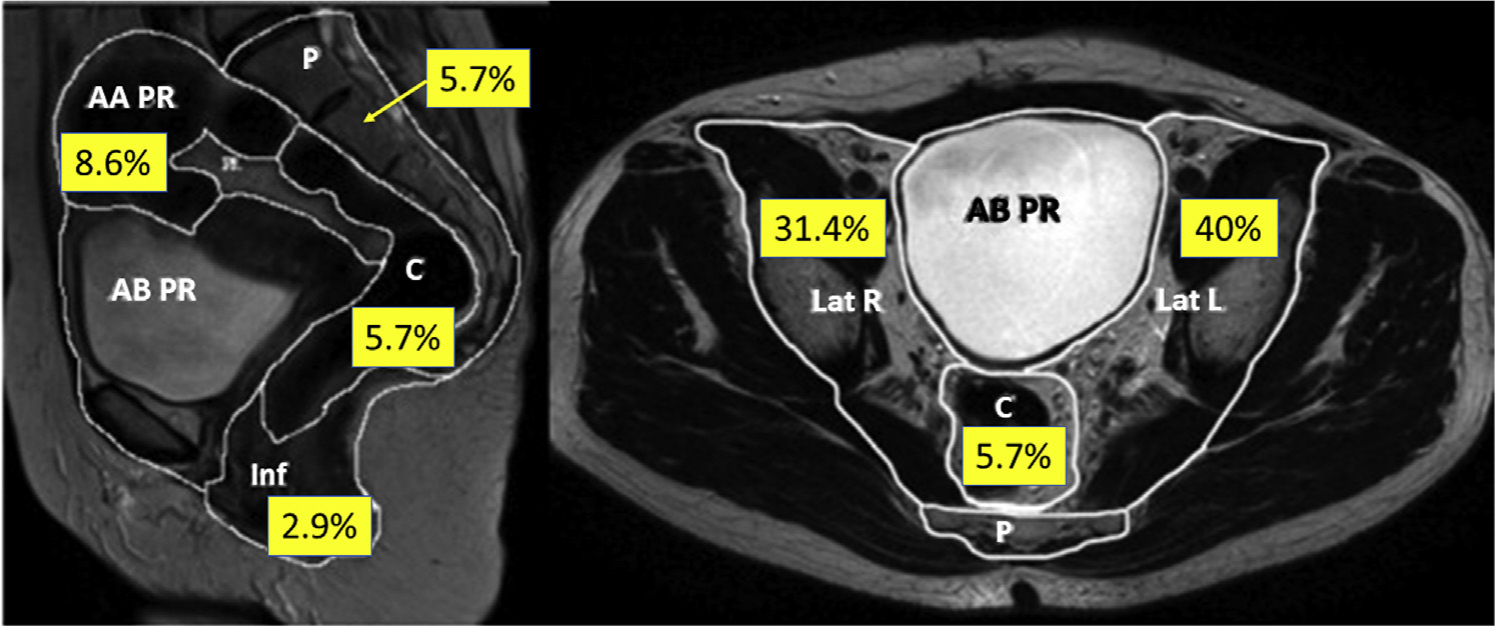
Distribution of targets within each pelvic compartment (N = 35). Two additional targets in the inguinal region, which co-occurred with another treated area inside the pelvis, are not shown (reproduced on permission from Georgiou et al^21^). *Abbreviations*: AAPR = anterior above peritoneal reflection; ABPR = anterior below peritoneal reflection; C = central; Inf = inferior; Lat L = lateral left; Lat R = lateral right; P = posterior.

The majority of patients had received prior chemotherapy, in either the adjuvant or metastatic setting, although 30% were chemotherapy naïve at the time of treatment and no chemotherapy was administered for the recurrence. Treated recurrences were located most frequently in the lateral compartments of the pelvis (71.4%; Fig. 2).

The most common (73%) prior radiation dose was 45 Gy in 25 fractions with concurrent capecitabine. The cumulative, median biologically effective doses with an alpha/beta ratio 5 and 10, along with cumulative equivalent dose in 2 Gy per fraction are shown in Table 1. No adjustment for recovery was made.

**Table 2:** Acute and late toxicity reporting

Toxicity	Acute N (%)	Late N (%)
Grade 1 (all)	7 (23)	
Pain	2 (6)	1 (3)
Fatigue	8 (26)	-
Proctitis	1 (3)	-
Cystitis	1 (3)	3 (10)
Grade 2 (all)	5 (16)	
Pain	1 (3)	-
Cystitis	1 (3)	-
Diarrhea	4 (13)	2 (6)
Grade 3 (all)	-	
Pain	-	1 (3)

Grading was as per Common Terminology Criteria for Adverse Events, version 4.0.

### Efficacy

**Figure 3:**
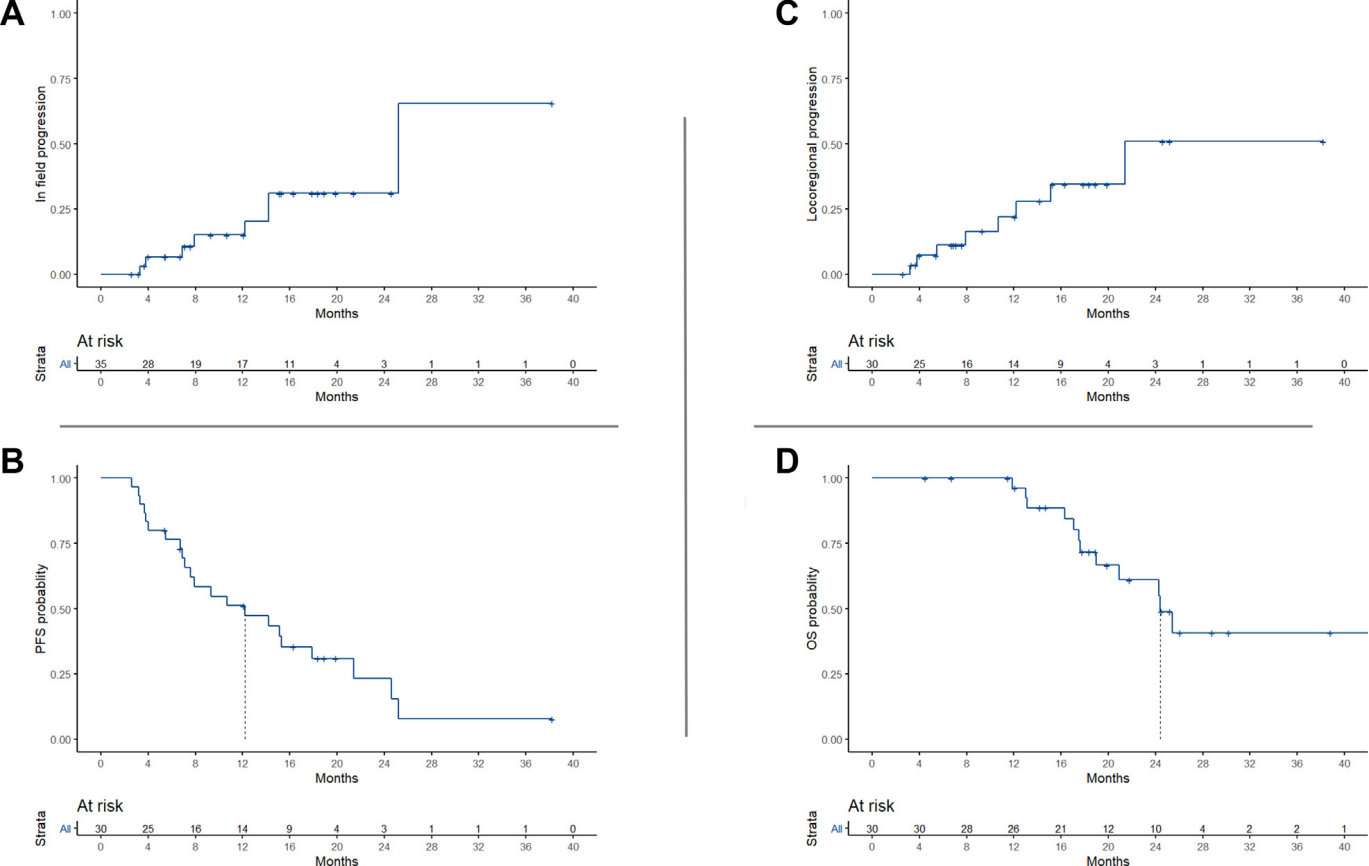
Kaplan-Meier estimates and associated risk tables of local control or in field progression. (A) Local control (N = 35); Loco-regional control (B) progression free survival; (C) and overall survival (D). Vertical dashed lines indicate median survival estimates.

Using Kaplan-Meier estimates, the 1-year local control was 84.9% (95% CI, 70.6-99) and a 2-year local control of 69% (95% CI, 51.8-91.9; Fig. 3A). Locoregional control at 1 and 2 years was 78% (95% CI, 62.3-97.7) and 49% (95% CI, 25.5-94.6), respectively (Fig. 3B). The median PFS was 12.1 months (95% CI, 8.6-17.66; Fig. 3C). The median OS was 28.3 months (95% CI, 17.88-39.5 months) with a 1-year OS of 95.0% and a 2-year OS of 84.4% (Fig. 3D).

### Quality of life outcomes

**Figure 4:**
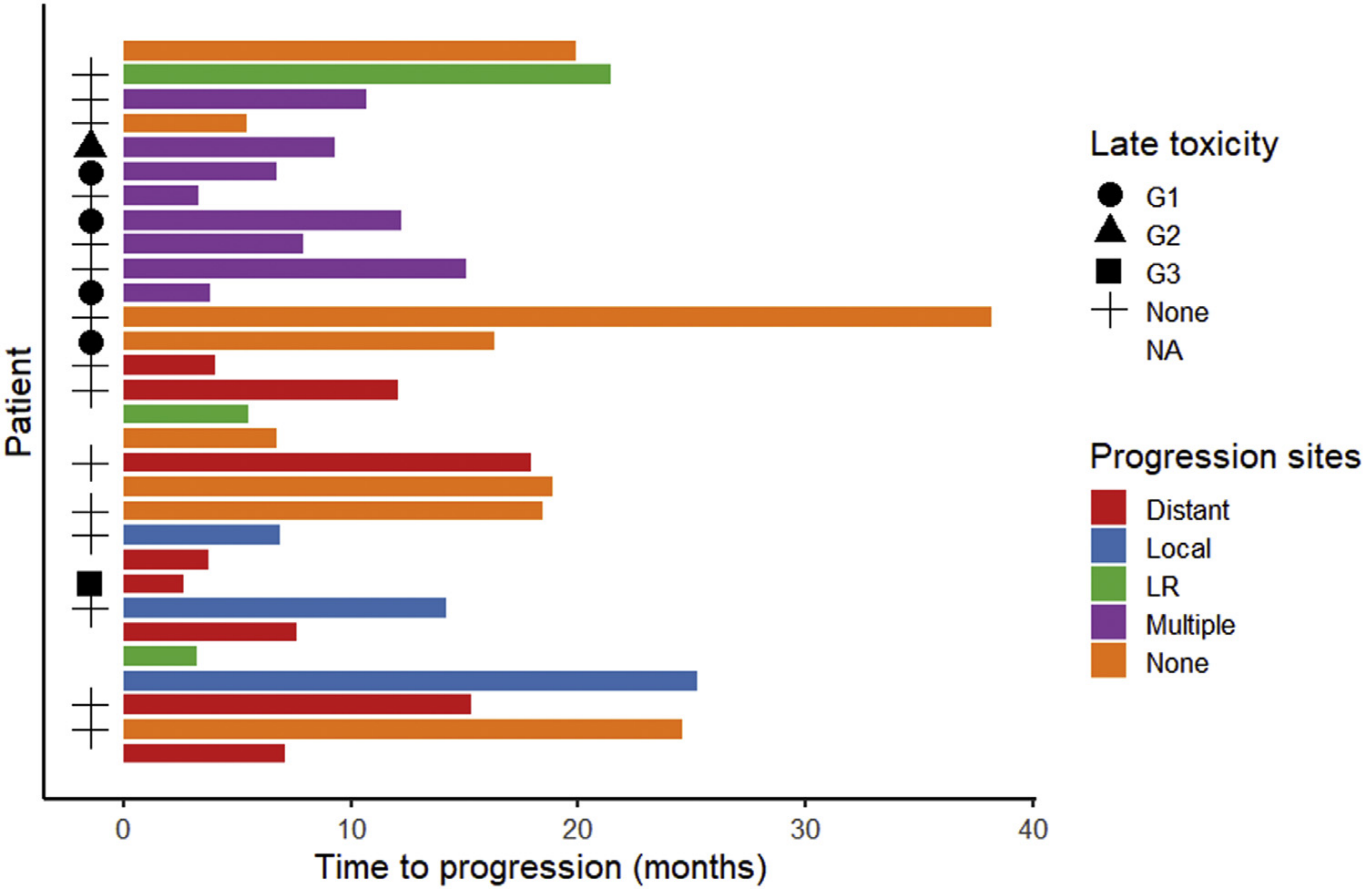
Swimmer plot of each patient showing time to progression and whether they progressed locally (local), locoregionally (LR), distant only (distant), at multiple sites (multiple), or had sustained local control (none). Development and grade of late toxicity is also shown.

Most patients were asymptomatic at presentation, with pain (28% G1/2) being the most common baseline symptom. Only one patient had baseline symptoms above Common Terminology Criteria for Adverse Events, version 4.0 G2, with G3 diarrhea. Acute toxicity data was available in 93% (28/30) of the cohort. The most reported acute toxicity was G1 fatigue (26.1%) and G2 diarrhea (13%), with no acute toxicities greater than G2 (Table 2). Late toxicity data was available for 80% of the cohort (24/30). One patient experienced G3 pain, possibly related to SBRT, which started approximately 15 months after completion of treatment, 12 months after distant relapse, and without clear evidence of local progression on serial imaging (Fig. 4). The most common late toxicity was G1 cystitis (10%). At the time of reporting, no patients had experienced symptomatic fractures of the pelvis, osteoradionecrosis or lymphedema which is encouraging given the large proportion of pelvic side wall disease treated. The median EQ-VAS score, at 3 months post reirradiation, improved from a median of 75 (range, 40-80) at baseline to 85 (range, 45-90; Wilcoxon signed-rank, *P* = .009).

## Discussion

Although advances in the multimodality treatment of rectal cancer have reduced local recurrence, it remains a significant clinical challenge. Our cohort adds to the current literature in a number of important ways; it is the largest SBRT cohort to date, patients were treated within the last 5 years, we used a prospectively maintained database with clear patient criteria and standardized treatment delivery. We demonstrate that reirradiation with 30 Gy in 5 fractions is well tolerated and achieves excellent local control rates with limited toxicity. We also report on patient reported outcome measure, which is entirely unique in this setting and absent from previous studies.

The local control rates 84.9% and 69% at 1-year and 2-years, respectively, compare favorably with previous reports of reirradiation in LRRC. A recent meta-analysis estimated 1 and 2-year LC of 72.0 % and 54.8% for patients treated with reirradiation alone, and 84.4% and 63.8% in patients who underwent reirradiation plus surgery.^10^ The median OS was 28.3 months (95% CI, 17.88-39.5 months), which again is superior to that seen in the previous reviews of reirradiation alone in LRRC, although other SBRT series also recorded high LC and OS.^17^^,^^19^^,^^25^ Whether this is a genuine increase in effectiveness or a selection bias toward smaller, better prognosis tumors is unclear. In a cohort of presacral recurrences with a median tumor volume of 52.5 cm, Heron et al^18^ demonstrated 1- and 2-year LC of 90.9% and 68.2.%, respectively with median dose prescription of 36 Gy in 3 fractions. No G3 or G4 toxicities were reported at a median follow-up of 16.1 months. Dagoglu et al treated 18 patients with a median dose of 25 Gy in 5 fractions, achieved an estimated 1- and 2-year LC rate of 100% and 93.7%, respectively with a median follow-up of 38 months. Approximately 60% of lesions were located in the pelvic side wall. Two G3 and one G4 toxicity were reported. Lastly, in the first SBRT series for LRRC reported, Kim et al demonstrated a 4-year local progression free survival rate of 74.3% for 23 patients treated with a median dose of 39 Gy in 3 fractions and reported one G4 toxicity.^19^ Despite clear heterogeneity within and between these cohorts, SBRT achieves high LC rates with acceptable toxicity rates.

Previous systematic reviews, which included SBRT studies, have demonstrated superior survival outcomes with surgery for LRRC, albeit at a higher risk of late toxicity.^6^^,^^10^ All patients in this cohort were assessed by a surgical service experienced in the management of LRRC before referral for SBRT. Location of recurrence is often the determining factor when assessing suitability for surgery with posterior or lateral tumors involving the sacral promontory, iliac vessels, or pelvic wall often contraindications for surgical resection,^7^^,^^26^ although surgeons may disagree on the criteria for case selection. It is therefore unsurprising that 71.4 % (25/35) of the lesions treated in this series were located where surgery is often contraindicated. Alternatively, this lateral pelvic side wall disease may not have been identified at the time of the primary treatment (radiation and surgery). Standard radiation approaches and total mesorectal excision are not always sufficient to prevent lateral lymph node recurrences in locally advanced lower rectal cancers, with a suggestion that lateral nodal dissection improves local recurrence rates.^27^ Primary diagnostic images were not available for clarification in the data set. In our opinion, the most likely clinical scenario is that of a seeded, radioresistant, clonal metastases at the treated site. All the treated sites were reviewed for position within the previous radiations fields and found to be in the high dose areas. Whether the clone was present in the primary rectal tumor, or at the metastatic site, during the initial radiation therapy course is unknowable. Given that all patients were thoroughly restaged before treatment and found to have only 1 to 2 lesions, would argue against seeding of a radiosensitive metastases from outside the previous RT field.

Several studies have sought to determine factors that may help identify which patients would be most likely to benefit from reirradiation.^13^^,^^19^^,^^25^^,^^28^ Tumor location and size are the most associated with differing treatment outcomes. Axial and anterior tumors are associated with more toxicity after reirradiation probably because of the increased dose to small bowel and bladder.^29^ In our cohort, 71.4% (25/35) of the targets were in the lateral pelvic compartments and could account for the low toxicity rates seen here. The importance of tumor size is less clear in terms of influencing high-grade toxicity^19^^,^^22^^,^^30^ with one study suggesting that tumors less than 3 cm in size may have better long-term control.^28^ The median gross tumor volume in this cohort was 14.3 cm^3^ (equivalent sphere 3 cm), smaller than previous SBRT cohorts, which could have contributed to the excellent local rates and OS.

The median OS is comparable to the median survival after R1 resection from the recent PelvEx Collaboration.^31^ This suggests that unless the preoperative likelihood of an R0 resection is high that SBRT is potentially viable alternative, accepting that the presented cohort may have an inherent better prognosis due to small volume disease compared with some resected cohorts. Reirradiation has the potential to delay the time to systemic treatment, provide local control and avoid unnecessary morbid surgeries for whom systemic disease becomes the life limiting issue. Surgeons may disagree on the “operability” of some of the treated cases underlining the need for a consensus approach to LRRC.

Concerns regarding reirradiation have focused on the potential for severe toxicity. Older series of reirradiation for LRRC demonstrated grade 3 or 4 acute toxicities rates of up to 35%.^11^^,^^12^^,^^32^^,^^33^ Clinical evidence dictating reirradiation tolerances of the bladder, small bowel, and other pelvic contents is lacking and no consensus dose constraints exist to date.^34^ Abusaris et al set cumulative constraints of <10 cm^3^ small bowel to receive no more than 110 Gy_3_ EQD2, <10 cm^3^ bladder to receive no more than 120 Gy_3_ EQD2 and, along with tight margins and high-quality image-guided radiation therapy, demonstrated low toxicity rates.^22^ Others suggest subtracting the previous dose from the traditional constraint, or, where not possible, they suggest that a degree of repair of up to 50% could be assumed depending on the interval to reirradiation.^16^ Using these approaches, we demonstrate a high local control rate with low late toxicity rates, with the majority (53%) reporting no acute toxicity and none greater than G2. The rapid dose fall-off generated by an SBRT means that point doses within 2 cm of the PTV are the main limiting factors in target coverage (Fig. 1C). This in turn is governed by individual patient anatomy and site of recurrences.

Proton radiation therapy (PRT) for reirradiation has demonstrated significant dosimetric advantages over IMRT in LRRC, with reduced small bowel doses.^35^ However, out of 7 patients, 3 had acute G3 toxicity and 3 had late G4 toxicity. The overall reirradiation mean PRT was 61.2 Gy and the mean clinical target volume was also significant larger at 246 cm^3^, which may have resulted in the increased toxicity. But these data underline that there is no “gold-standard” radiation modality for reirradiation with many patient and treatment related factors that need accounting for. Ultimately the goal for patients who are deemed inoperable is to achieve durable local control, minimal toxicity and sustained quality of life scores (QoL). The optimal way to achieve that goal-SBRT, PRT, hyperfractionation should be an active area of research.

We have demonstrated a statistically significant improvement in patient reported QoL scores at 3 months using the EQ-5D VAS at the end of the acute toxicity phase. The exact mechanism by which patients would report feeling better from SBRT is unclear. It is possible patients reported an improvement in a global health assessment scale owing to the psychologic benefit of “something being done.” However, it is clear that patients did not feel worse after the acute toxicity phase had resolved, which coupled with the low objective toxicity rates, suggests SBRT is well tolerated.

This study is limited by the small numbers of patients included, which although the largest to date, limits firm conclusions being drawn. The inherent nature of a nonrandomized study risks, selection bias and an overestimation of the efficacy of treatment. These risks are minimized by the a priori defined criteria for entry in the SBRT treatment program and the prospective nature of the data collection.

## Conclusions

Our findings demonstrate that SBRT reirradiation for patients who are nonsurgical candidates with small volume pelvic recurrence has minimal acute toxicity and offers the opportunity for local disease control and symptom control and improved QoL. We believe there is an imperative across the interested disciplines of radiation and surgical oncology to integrate existing knowledge and experience with a multidisciplinary approach to a clinical trial, to ask the relevant questions in LRRC.

## Supplementary Materials

Table E1
