# Supplementary material for: Stereotactic Body Radiation Therapy Reirradiation for Locally Recurrent Rectal Cancer: Outcomes and Toxicity
Source: Adv Radiat Oncol. 2020 Aug 7;5(6):1311–9. doi: 10.1016/j.adro.2020.07.017 (PMC7718547; doi:10.1016/j.adro.2020.07.017)
Supplement: Table E1 [file mmc1.docx]

**Supplementary Table 1.** Cumulative tolerances and dose constraints. Assumes a previous dose of 45Gy in 25 fractions and that maximum point doses were received by all OARs. An α/β of 3 is used to model late responding normal tissues

| **Organ at Risk** | **Previously delivered**  **[EQD_2_]** | **Cumulative tolerances [EQD_2_]** | **Remaining tolerance [EQD_2_]** | **Dose constraint**  **(5 fractions)** |
| --- | --- | --- | --- | --- |
| **Small Bowel D _0.5cc_** | 43.2 | 98 | 54.8 | < 30.3Gy |
| **Bladder D _0.5cc_** | 43.2 | 120 | 76.8 | <37 Gy |
| **Rectum D _0.5cc_** | 43.2 | 110 | 66.8 | <34 Gy |
| **Sacral nerves D _0.5cc_** | 43.2 | 60 | 16.8 | <14.4 Gy |
